# Conference Report on Public Health and Clinical Guidelines for Anthrax

**DOI:** 10.3201/eid1404.070969

**Published:** 2008-04

**Authors:** Eric Jacob Stern, Kristin Broome Uhde, Sean Vincent Shadomy, Nancy Messonnier

**Affiliations:** * Centers for Disease Control and Prevention, Atlanta, Georgia, USA

**Keywords:** anthrax, *Bacillus anthracis*, bioterrorism, guideline, pediatrics, prophylaxis, treatment, screening, conference summary

## Abstract

Conference Report on Public Health and Clinical Guidelines for Anthrax

On March 13–14, 2006, a meeting on anthrax, sponsored by the Centers for Disease Control and Prevention (CDC) in collaboration with the Southeastern Center for Emerging Biologic Threats, was held at Emory University in Atlanta, Georgia, USA. The meeting’s agenda included discussion of postexposure prophylaxis (PEP), screening and evaluation, and treatment of the various manifestations of human anthrax. The goal was to convene subject matter experts for a review of research developments and clinical experience with anthrax prophylaxis and treatment and to make consensus recommendations for updating guidelines for PEP, treatment, and clinical evaluation of patients with anthrax. A 2001 conference on guidelines for anthrax has previously been summarized in this journal (*1*). This article summarizes the meeting’s presentations and discussion. Consensus recommendations are summarized in the Table. Updated CDC guidelines for treatment and prophylaxis of anthrax will be published in detail in other CDC publications and are available on CDC’s website at http://www.bt.cdc.gov/agent/anthrax/index.asp.

**Table:** Summary of recommended modifications to Centers for Disease Control and Prevention guidelines for postexposure prophylaxis and treatment of anthrax

1. Ciprofloxacin and doxycycline are equally recommended as first-line oral antimicrobials for postexposure prophylaxis (PEP) in adult and pediatric patients.
2. Ciprofloxacin is recommended as the first-line oral antimicrobial for PEP and treatment in pregnant women; doxycycline should not be used during pregnancy for PEP or treatment until the third trimester.
3. Ciprofloxacin is favored over doxycycline for treatment of anthrax cases with serious systemic illness such as: inhalation anthrax; gastrointestinal anthrax; cutaneous anthrax with systemic involvement; cases with suspected meningeal involvement; and fulminant cases with bacteremia.
4. Meningeal involvement should be suspected in inhalation anthrax or in any case of systemic anthrax, therefore treatment should consist of intravenous therapy with ciprofloxacin plus one or two additional antimicrobial agents with adequate CNS penetration and proven efficacy against B. anthracis.
5. The use of early, aggressive serial or continuous drainage of pleural effusions is recommended for all inhalation anthrax cases.

Participants included representatives and members of academic research and clinical institutions, the Health Protection Agency of the United Kingdom, the Health Protection Agency and Armed Forces of Canada, the US Department of Defense, the US Department of Homeland Security, the US Department of Health and Human Services Office of Research and Development Coordination, the Food and Drug Administration, the National Institutes of Health, the Council of State and Territorial Epidemiologists, the American Board of Obstetricians and Gynecologists, the Infectious Diseases Society of America, and CDC (Figure). Among these participants were researchers, health department personnel, and clinicians, including the Pennsylvania pulmonologist who treated the 2006 case of inhalation anthrax (IA), the first naturally occurring case of IA in the United States since 1976 (*2*).

**Figure:**
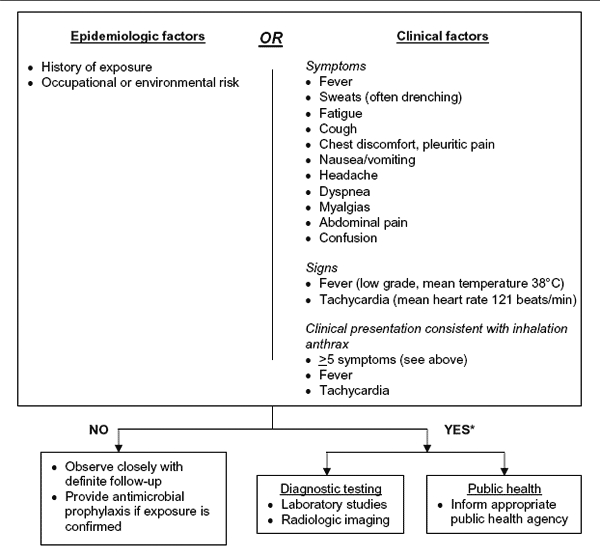
Revisions to the Centers for Disease Control and Prevention (CDC) interim inhalation anthrax screening guidelines proposed by Mayer et al (*29*), and reviewed by participants in CDC meeting on public health and clinical guidelines for anthrax. For further detail on subsequent algorithm steps see (*29*). Adapted from (*29*).

## PEP for Inhalation Anthrax

During the 2001 US outbreak of bioterrorism-related anthrax, 22 confirmed or suspected anthrax cases occurred after envelopes containing *Bacillus anthracis* spores in powder were sent through the mail (*3*). Approximately 10,000 persons were offered at least 60 days of antimicrobial PEP (*4*). Current CDC recommendations for PEP following potential inhalation exposure to aerosolized *B. anthracis* spores are 60 days of oral antimicrobial therapy in combination with a 3-dose series of anthrax vaccine adsorbed (AVA), BioThrax (BioPort Corporation, Lansing, MI, USA) administered at time zero, 2 weeks, and 4 weeks (*5**,**6*). AVA is not FDA-approved for PEP and therefore would be available under an Investigational New Drug (IND) protocol.

### Antimicrobial Agent Selection

Selection of the antimicrobial agent for PEP should involve consideration of antimicrobial resistance. Variable β-lactam resistance, particularly to the cephalosporins, has been reported among naturally occurring *B. anthracis* isolates (*7**–**9*). β-lactamase genes have been identified in the *B. anthracis* chromosome (*10**,**11*), and evaluation of isolates from the 2001 event indicated the presence of both cephalosporinase and penicillinase enzymes (*12*). Additionally, resistance can be readily induced in vitro in *B. anthracis* to a variety of antimicrobial classes including fluoroquinolones, tetracyclines, macrolides, penicillins, and other β-lactams (*8**,**13**–**16*).

Ciprofloxacin, doxycycline, and penicillin G procaine have demonstrated efficacy for PEP in a nonhuman primate model (*17*) and are FDA-approved for “inhalational anthrax (postexposure)” in all age groups (*18*). Meeting participants reiterated existing CDC recommendations for ciprofloxacin and doxycycline as equivalent first-line antimicrobial agents for PEP, as they are equally efficacious for PEP and have similar susceptibility profiles among naturally occurring isolates (*9**,**17*). Both have similar safety profiles, with a low rate of anaphylactic reactions (*19**,**20*). Following the bioterrorism event of 2001, there were no differences in self-reported symptoms with use of either drug for PEP, and no serious adverse events could be definitely related to their use (*21*).

Levofloxacin is FDA-approved for “inhalational anthrax (postexposure)” in adults 18 years of age and older (*22*). There are safety data for up to 28 days of use, but safety data on extended use up to 60 days are limited (*22*). Therefore, levofloxacin is recommended as a second-line PEP antimicrobial agent, to be reserved for instances where medical issues may call for its use.

Penicillins should not be initially used for PEP of anthrax, due to concern for penicillin resistance, which has been found in naturally occurring isolates, and because of the low concentrations achieved with oral penicillins in pulmonary secretions, tissue, and within alveolar macrophages (*23**,**24*). Amoxicillin can be used for PEP once the *B. anthracis* strain has been proven penicillin susceptible, when other antimicrobial agents are not considered safe to use, such as for pediatric patients and for nursing or pregnant women. However, amoxicillin is not FDA-approved for this indication, and this use is considered “off-label.” Therefore, amoxicillin use for PEP in a mass-exposure event might be provided under an IND or under an Emergency Use Authorization in a declared emergency. Amoxicillin use for PEP is discussed further in the section below on special populations. Other antimicrobial agents, including clindamycin, chloramphenicol, rifampin, vancomycin, and other fluoroquinolones, may be considered for off-label use in patients unable to tolerate FDA-approved antimicrobial agents for PEP.

### Duration of Prophylaxis Regimen

The PEP antimicrobial regimen should remain for 60 days, combined with 3 doses of AVA. This duration is supported by data from the 1979 Sverdlovsk anthrax outbreak (*25*): illness up to 58 days following inhalation exposure to anthrax in nonhuman primates receiving antibiotics alone for 30 days (*17*), and demonstrated efficacy of the PEP combination of antimicrobial therapy and vaccination with AVA (*17*).

Participants discussed whether a shortened course of antimicrobial therapy plus a 3-dose AVA series would be effective, based on recent nonhuman primate research of a successful 14-day PEP course of ciprofloxacin combined with 3 AVA doses, and demonstrated immune response to AVA (*26*), and on evidence of seroconversion among clinical trial participants following 3 doses of AVA (*27*). However, there are no well-defined serologic correlates of protection to demonstrate that AVA vaccination has conferred adequate protective immunity in a person receiving PEP. Additionally, there are no human clinical trial data supporting any reduction in the duration of antimicrobial PEP, and there are isolated AVA study participants who have failed to seroconvert following vaccination (CDC, unpub. data). Therefore, it was deemed prudent to maintain the recommended 60-day course of antimicrobial therapy combined with the 3-dose AVA series to ensure adequate protection for all persons requiring PEP after aerosolized *B. anthracis* exposure.

## Clinical Screening and Evaluation of Inhalation Anthrax

Since 2001, when CDC guidelines for the clinical evaluation of patients with possible IA in the event of possible mass exposure were published in the MMWR (*28*), several alternative screening algorithms have been suggested including the Inova Fairfax protocol proposed by Mayer et al (*29*), and the 3-tier screening protocol from Hupert et al (*30*).

The goal of all IA screening algorithms should be to evaluate large numbers of patients seeking treatment in emergency departments and identify potential IA cases: 1) during a potential or confirmed mass event; 2) when there is a known threat or epidemiologic data suggesting an increased risk for anthrax; or 3) when there is clinical suspicion of anthrax based on symptoms consistent with IA, including fever and persistent tachycardia, among others (*28**,**29*). The low sensitivity and specificity of such screening algorithms may not detect an isolated IA case; therefore, these algorithms are not meant to serve as general guidance for identifying IA without appropriate epidemiologic or clinical data. For example, neither the original CDC guidelines nor the proposed Inova Fairfax guidelines would have detected the solitary 2006 IA case (*2*). Nor should IA screening algorithms replace existing emergency department (ED) screening guidelines for patients with symptoms of community-acquired pneumonia (CAP); routine CAP cases are likely to be selected as potential IA cases when using these algorithms; additional diagnostics will be required to rule out IA.

Mayer et al retrospectively evaluated CDC’s screening guidelines, using the 11 IA cases from 2001 included among an ED patient population from the same period. On the basis of their analysis, they proposed revised screening guidelines. CDC guidelines successfully identified only 1 of the 11 cases, whereas the Inova Fairfax algorithm modified from CDC guidelines successfully identified 9 of the 11 cases (*29*). Participants debated whether the recommendation to reduce the number of clinical symptoms required by the Inova Fairfax guidelines warranted further evaluation (Table) because IA case-patients who seek treatment early in the course of the disease could be missed as they may not yet have all of the required symptoms. Including fever and tachycardia as necessary symptoms to begin the algorithm, timing public health notification, and reducing the stringency of the requirement for an epidemiologic link were discussed. Participants agreed that elements of the Inova Fairfax protocol should be incorporated into future CDC-recommended screening algorithms. They further recommended that validation studies be conducted and feedback collected to determine the accuracy and effectiveness of the algorithm in identifying cases of IA in an outbreak setting.

Hupert et al proposed a 3-tier screening protocol to identify potential early IA case-patients in the setting of a large-scale anthrax exposure, to aid ED physicians in decisions regarding PEP, and to support the clinical treatment decision process (*30*). Participants agreed that such a large-scale screening algorithm for persons with potential aerosol exposure to anthrax in a mass exposure setting should be addressed in CDC’s guidelines; however, such an algorithm was not adequately validated for adoption as a CDC recommendation.

Clinicians considering IA as a differential diagnosis should alert hospital microbiology staff of their suspicions and obtain blood cultures as early as possible before antimicrobial treatment. Gram-stain analyses of blood samples have previously detected bacteremia in systemic anthrax cases and in animal models (*31**,**32*) and may be informative.

Thoracic imaging remains a critical tool for the diagnosis of IA. Thoracic imaging studies were abnormal in all 11 of the IA cases from 2001 (8/11 with widened mediastinum, 9/11 with pleural effusions, and 7/11 with pulmonary infiltrates). However, initial ED thoracic radiographs may not reveal the classic widened mediastinum described for IA in all cases (*33**,**34*). In severe IA cases, thoracic computerized tomography without contrast was suggested as having utility for viewing hemorrhagic mediastinal lymph nodes.

Sensitivity and specificity analyses were recommended for evaluating any proposed IA screening algorithms, using historical IA cases incorporated into ED populations from annual influenza seasons or other periods with increased numbers of respiratory illness cases. Neither current CDC nor proposed alternative guidelines are applicable for pediatric patients because of lack of data on pediatric IA cases. Participants called for development of screening guidelines for children in collaboration with the American Association of Pediatrics and other pediatric care partners.

### Treatment of Severe Disease, including Inhalation and Gastrointestinal Anthrax, Anthrax Meningitis, and Bacteremia

Current CDC recommendations for the treatment of anthrax were published in October 2001 specifically for cases resulting from the 2001 anthrax bioterrorist attack. After presentations on the clinical course of IA cases from 2001 and 2006 and discussion of the limited treatment success of IA cases from 2001 (5/11 patients died despite aggressive therapy), participants recommended revision of CDC treatment protocols for IA and serious systemic illness from anthrax.

Clinical or subclinical meningitis in patients with IA is likely. Meningitis is reported with all 3 clinical forms of anthrax and likely results from hematogenous spread across the blood-brain barrier when bacteremia is present. During the 2001 outbreak, although only 1 of the 11 IA case-patients had meningitis, 4 others had symptoms suggesting meningeal involvement. Confirmation of meningitis was not obtained in these 4 case-patients, and cerebrospinal fluid was not examined in all patients. If these 4 case-patients did have subclinical or early meningitis, then 45% of the 2001 IA case-patients had meningeal involvement (*33*). Additionally, hemorrhagic leptomeningitis was reported from autopsies in 21 (50%) of 42 IA fatalities from the 1979 Sverdlovsk outbreak (*35*); meningoencephalitis was reported in 44% of fatal cases in a review of 82 IA cases from 1900 to 2005 (including the 11 bioterrorism (BT)-related cases from 2001) (*34*). Furthermore, IA studies of nonhuman primates have demonstrated meningeal involvement in up to 77% of experimental animal cases (*32*). Therefore, meningeal involvement should be suspected in IA or other cases of systemic anthrax.

For treatment of anthrax cases with severe systemic or life-threatening disease (including IA and gastrointestinal anthrax), and for cases with fulminant bacteremia, IV ciprofloxacin is recommended over doxycycline as the primary antimicrobial agent unless ciprofloxacin use is contraindicated (fluoroquinolones are bactericidal while tetracyclines are bacteriostatic). Additionally, because meningeal involvement is likely in systemic anthrax cases, ciprofloxacin is theoretically favored over doxycycline; central nervous system (CNS) penetration of ciprofloxacin in the presence of meningeal inflammation is much higher than the poor CNS penetration of doxycycline (*31*). Although ciprofloxacin is the only fluoroquinolone for which data are available, other fluoroquinolones with similar spectrums of activity and CNS penetration may also be appropriate.

At least 1 or more additional agents with adequate CNS penetration and in vitro activity against *B. anthracis* (e.g., ampicillin or penicillin, meropenem, rifampin, or vancomycin) should be used in the treatment of systemic cases of anthrax regardless of clinical suspicion of meningeal involvement. Clindamycin is strongly recommended for inclusion in the antimicrobial regimen because of its ability to inhibit protein synthesis, which may reduce exotoxin production. Participants recommended continuing the current 60-day course of antimicrobial therapy, with adjustment of the regimen based on the clinical course of the disease in the patient (*36*). The use of corticosteroids as an adjunct to antimicrobial therapy may benefit patients with anthrax meningitis (*31*); however, with no efficacy data from controlled clinical trials, this adjunctive treatment may be of no benefit for toxin-mediated tissue edema.

Early and aggressive pleural fluid drainage is recommended for all IA case-patients and is consistent with the standard of care for empyema or complicated pneumonia. This recommendation is based on the experience that chest tubes or early serial drainage of pleural effusions seemed to be beneficial in the successful clinical therapy of the surviving IA patients in 2001 and in the recent 2006 IA case (*2**,**33*). Evaluation of the treatment of IA cases from 1900 to 2005 showed pleural fluid drainage to be significantly associated with decreased mortality (*34*). Analysis of serial pleural fluid samples from the 2006 IA case showed high pleural fluid lethal toxin levels. The positive outcome of this case likely resulted from a combination of the mechanical effects on respiration from fluid drainage and the reduction in lethal toxin levels by removing pleural effusions (*2*).

## Treatment of Cutaneous Anthrax

Treatment with oral ciprofloxacin or doxycycline for 7–10 days is recommended for localized or uncomplicated cases of naturally acquired cutaneous anthrax, such as that associated with exposure to animals with anthrax, or to products such as hides from animals with anthrax. If susceptibility testing is supportive, oral therapy with penicillin V or amoxicillin may be used to complete the course of treatment. For severe cases of naturally acquired cutaneous anthrax with signs of systemic involvement, extensive edema, or lesions of the head and neck, IV therapy for 7–10 days using ciprofloxacin or doxycycline is preferred; IV penicillin G, if supported by susceptibility testing, may be used to finish the 7- to 10-day course. It was considered widely accepted knowledge that penicillin is very effective against *B. anthracis*, can render cutaneous lesions culture-negative within 24 hours, and has long been the treatment of choice in many parts of the world. Therefore, despite reports of penicillin-resistant isolates and the potential for inducible beta-lactamases, participants agreed that 7–10 days of penicillin is usually sufficient for treatment of uncomplicated naturally acquired cutaneous anthrax. However, adequate dosages of penicillins must be used, patients must be monitored for clinical response, and susceptibility testing must be conducted to confirm the appropriateness of the antimicrobial choice. Other fluoroquinolones such as levofloxacin are recommended as additional options for antimicrobial treatment of naturally acquired cutaneous anthrax.

For bioterrorism (BT)-related cutaneous anthrax, any patient is at risk for IA because of potential aerosol exposure; the duration of antimicrobial therapy should remain 60 days to provide a full course of PEP. For localized or uncomplicated cases of BT-related cutaneous anthrax, oral ciprofloxacin or doxycycline is recommended. Patients with BT-related cutaneous anthrax with signs of systemic involvement, extensive edema, or lesions of the head or neck should be treated with IV ciprofloxacin using a multidrug approach, as previously described for IA and severe disease. Patients may be switched to oral therapy when clinically appropriate to complete the 60-day regimen; in uncomplicated cases amoxicillin may be used to complete the regimen if the *B. anthracis* strain is proven susceptible (*36*).

There is insufficient evidence to conclude that the immune response to cutaneous anthrax is sufficient to justify shortening the 60-day duration of antimicrobial therapy. However, severe local reactions to anthrax vaccine were reported in 2 vaccine recipients with a previous history of cutaneous anthrax during a trial of an earlier protective antigen-based anthrax vaccine (*37*). For BT-related cutaneous anthrax in patients without a prior history of cutaneous disease, the recommended regimen should remain 60 days of antimicrobial therapy plus 3 doses of AVA; for BT-related cutaneous anthrax in patients with a previous history of cutaneous disease, 60 days of antimicrobial therapy is recommended but vaccine should not be used because of the risk of adverse events (*37*).

### Immunotherapeutics

Immune globulin of animal origin has been used with some success in the treatment of human anthrax for many years (*34**,**38*), and human-derived anthrax immune globulin (AIG) was part of the successful treatment of the 2006 IA case-patient under an emergency IND protocol. (*2*). Although participants agreed that clinical data are insufficient to develop general recommendations for the use of AIG and other targeted immunotherapeutics for the treatment of severe anthrax, they did recommend that CDC continue to offer AIG under emergency IND on a patient-by-patient basis.

Timing of immune globulin administration is critically important given the small window in which AIG can be effective as anthrax toxins bind and move intracellularly quickly; early administration to prevent toxin binding was stressed. However, data on the optimal timing to initiate immune therapy are lacking. Immunotherapeutics should continue to be considered for patients with severe systemic illness, for example in patients with evidence of organ dysfunction in >2 organ systems or lack of clinical response to standard therapy. A high research priority should be to continue to evaluate immunotherapeutics as they are developed for inclusion in future recommendations.

### Special Populations

Treatment of anthrax during pregnancy should remain the same as for adults because of the severity of the disease. Ciprofloxacin is recommended as the first-line oral antimicrobial agent for PEP or treatment of anthrax during pregnancy (*39*). Doxycycline should not be used for PEP or treatment during pregnancy unless started in the third trimester. Transition to amoxicillin for PEP remains recommended when the isolate involved is susceptible to penicillin.

Although the Department of Defense has published safety data on AVA during pregnancy (*40*), the statistical power of the study was limited. Meeting participants indicated that additional safety data are needed to support appropriate decisions regarding recommendations for this population. It was proposed that AVA be included in the PEP protocol for pregnancy if there is strong evidence of risk for IA or if the benefit outweighs risks from vaccination. Vaccination may also be deferred to immediately postpartum if the risk of IA persists because of limited safety data on AVA use during pregnancy.

For breastfeeding mothers, if the infant was also exposed, the mother’s antimicrobial regimen should match the child’s regimen when possible. When not possible, the mother can pump and discard her breast milk while being treated, and resume breastfeeding after completing her course of PEP.

Recommendations for treatment of BT-related anthrax in pediatric patients should be consistent with adult antimicrobial agents, with ciprofloxacin favored for severe disease. Ciprofloxacin or doxycycline is recommended as the first-line oral antimicrobial agent for PEP. Amoxicillin is recommended as an alternative in pediatric treatment when the isolate involved is susceptible to penicillin. Participants discussed the need to determine the appropriate amoxicillin PEP dose for pediatric patients to resolve differences between CDC and FDA recommendations. The consensus was that the amoxicillin PEP dose should err on the higher end in favor of ensuring efficacy. Data are lacking on the safety of long-term use of high-dosage amoxicillin in pediatric patients; participants recommended additional research and Pediatric Advisory Committee input to determine the most appropriate pediatric amoxicillin PEP dosage. Currently, anthrax vaccination cannot be recommended for use in children because safety and efficacy data are lacking.

Finally, for special populations such as geriatric patients or patients with special medical conditions, standard medical practice should prevail. Physicians should perform standard medical screening for potential drug interactions and for underlying diseases such as renal failure before initiating treatment or PEP; drug selection and dosage should be adjusted accordingly.

Research into the evaluation, prevention, and therapy of anthrax is ongoing, and CDC guidelines for anthrax are subject to change based on new information and expert recommendation. When available, updated CDC guidelines for treatment and prophylaxis of anthrax will be published in detail in other CDC publications.
